# Diagnostic value of RASSF1A methylation for breast cancer: a meta-analysis

**DOI:** 10.1042/BSR20190923

**Published:** 2019-06-28

**Authors:** Mingyi Li, Chunpeng Wang, Binbin Yu, Xueyuan Zhang, Fang Shi, Xin Liu

**Affiliations:** 1 Department of Epidemiology and Biostatistics, School of Public Health, Jilin University, Changchun 130021, Jilin, China; 2 School of Mathematics and Statistics, Northeast Normal University, Changchun 130000, Jilin, China

**Keywords:** breast cancer, diagnosis, DNA methylation, meta-analysis, RASSF1A

## Abstract

Background: Numerous studies reported that RAS-association domain family 1 isoform A (*RASSF1A*) methylation might act as diagnostic biomarker for breast cancer (BC), this meta-analysis aimed to evaluate the value of *RASSF1A* methylation for diagnosing BC.

Methods: Such databases as PubMed, Cochrane Library and Web of Science databases were searched for literatures until May 2019. A meta-analysis was performed utilizing STATA and Revman softwares. Furthermore, subgroup analysis was adopted to determine likely sources of heterogeneity.

Results: Totally 19 literatures with 1849 patients and 1542 controls were included in the present study. Sensitivity, specificity, diagnostic odds ratio (DOR) and the area under the summary receiver operating characteristic curve (AUC) of *RASSF1A* methylation for diagnosing BC were 0.49, 0.95, 19.0 and 0.83, respectively. The sensitivity (0.54 vs 0.43), DOR (30.0 vs 10.0) and AUC (0.84 vs 0.81) of *RASSF1A* methylation in Caucasian were higher than other ethnicities. The sensitivity (0.64 vs 0.57), DOR (21.0 vs 14.0) and AUC (0.89 vs 0.86) of methylation-specific PCR (MSP) were superior to other methods (q-MSP, OS-MSP and MethyLight). The sensitivity, DOR and AUC of serum *RASSF1A* methylation vs *RASSF1A* methylation in other samples (tissue or plasma) were 0.55 vs 0.40, 22.0 vs 14.0 and 0.86 vs 0.74, respectively.

Conclusions: *RASSF1A* methylation might be a potential diagnostic biomarker for BC. Considering its low sensitivity and high specificity, it should combine with others to upgrade the sensitivity. Besides, under such conditions, MSP detection, serum *RASSF1A* methylation and Caucasian are shown to be more effective and suitable for diagnosing BC.

## Introduction

Breast cancer (BC) is the most popular cancer and the most common reason for cancer death in female worldwide [1]. The development of breast cancer is a multi-step procedure, and its prevention is still a difficult task across the world. It is well known that early diagnosis is beneficial, in some advanced countries, the 5-year relative survival rate for BC patients is beyond 80% as a result of early diagnosis. Thus, early screening of BC among high-risk subjects represents a significant way to upgrade prognosis of the patients [2]. It is general practice among primary care, gynecology and oncology physicians as well as surgeons to carry out clinical examination when BC is suspected. Recent research reports numerous diagnostic biomarkers, but there is no clinical guideline or unanimous consensus with regards to the use of biomarkers for early diagnosis of BC [3]. Therefore, effective biomarkers are truly needed.

Many studies currently demonstrate that the detection of methylated circulating cell-free DNA in patient’s peripheral blood might be a favorable quantitative and non-invasive method for diagnosing BC [4]. The promoter region methylation of tumor suppressor genes causes transcriptional silencing. Many significant genes that suffer from transcriptional silencing are involved in critical cancer-related cellular pathways. Studies demonstrate that the patterns of gene methylation are distinctive and the abnormal methylation of genes could act as diagnostic biomarkers for BC [5].

RAS-association domain family 1 isoform A *(RASSF1A*), belonging to family of RAS effectors, is a tumor suppressor gene coding particular protein. *RASSF1A* is expressed in normal breast cells while inactivated in breast tumors due to DNA methylation of their CpG island in promoters, *RASSF1A* displays high promoter methylation of 56% in BC but 8% in normal tissues. The result suggests that DNA methylation of *RASSF1A* may be common in BC [6,7].

Increasing evidence shown the relationship between *RASSF1A* and BC, but the value of *RASSF1A* methylation in the diagnosis of BC is uncertain [8]. Mohammad et al. reported that the sensitivity of *RASSF1A* methylation was 32%, but Noriaki et al. showed the sensitivity was 90% [9,10]. The heterogeneity was high among different studies. In the present study, we used a meta-analysis to identify the diagnostic value of *RASSF1A* methylation for BC.

## Materials and methods

### Search strategy and study selection

Using a detailed search in PubMed, Cochrane Library and Web of Science, all related studies published up to May 2019 were retrieved. Search words were determined as follows: (*RASSF1A* methylation or hypermethylation) and (Breast Neoplasm or Neoplasm, Breast or Breast Tumors or Breast Tumor or Tumor, Breast or Tumors, Breast or Neoplasms, Breast or Breast Cancer or Cancer, Breast or Mammary Cancer or Cancer, Mammary). A comprehensive search strategy was displayed in Supplementary Table S1. The references of all related papers were checked for potential studies.

Two independent reviewers screened the literatures in line with the inclusion and exclusion criteria. The inclusion criteria included: (1) The research must investigate the diagnosis value of *RASSF1A* methylation for BC. (2) The patients were diagnosed with BC by pathology. (3) Literatures were published in English. (4) Sensitivity and specificity of *RASSF1A* were offered abundantly to build 2 × 2 contingent tables. The exclusion criteria were made as follows: (1) Studies were categorized as reviews, letters, guidelines, consensus statements, meeting abstracts and words of editors. (2) Studies lacked enough data, such as the *RASSF1A* methylation could not be extracted or computed from the original research.

### Data extraction and quality assessment

Two reviewers extracted following data from the eligible literatures: first author, year of publication, sample size, sample type, detection method and ethnicity. When disagreement was presented, a consensus was realized after discussion with a third reviewer. Quality Assessment of Diagnostic Accuracy Studies (QUADAS-2) was utilized to evaluate the quality of studies [11]. QUADAS-2 represented an evidence-oriented quality assessment approach including four areas: patient selection, index test, reference standard, and flow and timing.

### Trial Sequential Analysis (TSA)

Trial Sequential Analysis (TSA) was performed by the TSA software (Centre for Clinical Intervention Research, Copenhagen Trial Unit). Two-sided type I error of 5% and two-sided type II error of 5% were used in conventional test and α-spending boundaries. ‘Information size’, ‘Relative risk reduction’ and ‘Incidence in Intervention Group’ were automatically generated by the TSA software, and ‘Incidence in Control Group’ = 5% were set to calculate the required sample size. If the cumulative *Z*-curve crossed the monitoring boundaries constructed by both information size calculations and reached the required sample size line, the number of samples included in the meta-analysis is sufficient and the result is reliable.

### Statistical analysis

First, we measured the threshold effect and heterogeneity. If the Spearman correlation coefficient’s *P*-value was over 0.05, there was no threshold effect, then a heterogeneity produced through non-threshold effect possessed a further analysis. If *P* < 0.05 or *I*^2^ > 50%, the heterogeneity was significant and a random-effect model was built. Subgroup analysis were performed to find the sources of heterogeneity. The four covariates for subgroup analysis were as follows: (1) ethnicity of patients, (2) sample size, (3) the detection method and (4) the sample type. When *P* exceeded 0.05 and *I^2^* was not higher than 50% in one subgroup, the related covariate was seen as the source of heterogeneity. The pooled sensitivity, specificity, positive likelihood ratio (PLR), negative likelihood ratio (NLR) and diagnostic odds ratio (DOR), along with their 95% confidence intervals (Cls) were calculated and graphically shown in forest plots. A summary receiver operating characteristic (SROC) curve was built and the area under the SROC curve (AUC) was calculated to estimate the diagnostic performance of *RASSF1A* methylation. We utilized the Deek’s funnel plot to evaluate publication bias.

## Results

### Search results

**Figure 1:**
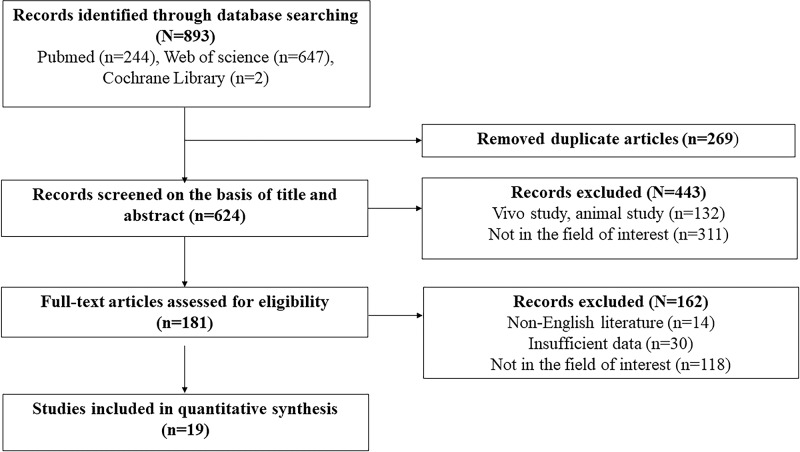
Flow diagram of the study selection process

A total of 893 literatures were searched from PubMed, Cochrane Library and Web of Science databases. No new literature was observed after looking for the references. Altogether, 874 literatures were excluded as they did not satisfy the inclusion criteria: 269 literatures were excluded owing to duplication, 443 literatures were excluded after screening the title and abstract; 162 literatures were excluded after assessing the full-text, Finally,19 literatures were included in this meta-analysis (Figure 1). Three literatures analyzed two different studies and eventually 22 studies were included in the meta-analysis.

### Study characteristics

The 19 proper articles [6–10,12–25] contained 1849 patients and 1542 controls in total. There were 8 articles based on serum [9,10,12,13,16,22,24,26], 10 articles based on tissue [6–8,15,17–19,25–27] and 4 articles based on plasma [14,18,21,28]. Six studies used q-MSP (quantitative methylation specific PCR) [14,15,17,18,24,26], 1 study used OS-MSP (the one-step methylation-specific polymerase chain reaction) [10], 3 studies used MethyLight (high-throughput DNA methylation assay) [19,22,25], 8 studies taken MSP [6,8,9,12,13,16,21,28] as the detection method of *RASSF1A* methylation and 1 study didn’t show methods for *RASSF1A* methylation. The patients’ ethnicity in 11 studies was Caucasian [6,12,13,15,18–22,24,26], 7 studies was Asian [8–10,16,24,25,28] and 1 study was African [14]. Six studies of sample size <100 [13,18–21,26] and 13 studies of sample size ≥100 [6,9,12,15–18,22,25,27,28,30,31]. More characteristics of included studies are shown in Table 1.

**Table 1:** Major characteristics of included studies

Author	Year	Tp	Fp	Fn	Tn	Sample size	Sample	Method	Ethnicity
Jovana, K.	2004	174	0	18	6	198	Tissue	q-MSP	Caucasian
Mohammad, O.H.	2006	15	2	32	36	85	Plasma	q-MSP	Africa
Eirini, P.	2006	13	0	37	14	64	Plasma	MSP	Caucasian
Feng, J.	2010	37	3	13	47	100	Serum	MSP	Asian
Joheon, K.	2010	39	6	80	119	244	Serum	q-MSP	Asian
Yoon, N.	2010	33	23	7	4	67	Tissue	MethyLight	Caucasian
Jennifer, D.	2010	11	28	39	119	197	Serum	q-MSP	Caucasian
Noriaki, Y.	2011	85	2	9	51	147	Tissue	OS-MSP	Asian
Nasser, S.R.	2013	17	0	19	14	50	Serum	MSP	Caucasian
Dominique, T.	2013	23	11	31	35	100	Tissue	MSP	Caucasian
Hoda, A., I	2013	84	3	36	97	220	Tissue	MSP	Caucasian
Hoda, A., II	2013	76	1	44	99	220	Serum	MSP	Caucasian
Mary, J.	2013	13	2	6	26	47	Tissue	q-MSP	Caucasian
Samia, A.E.	2015	50	0	30	80	160	Serum	MSP	Caucasian
Jolien, S.	2015	8	3	44	46	101	Tissue	q-MSP	Caucasian
Ming, S.	2016	46	25	222	437	730	Serum	MethyLight	Asian
Antje, M.	2016	15	2	12	23	52	Tissue	—	Caucasian
Zhong, H.H., I	2017	33	6	67	20	126	Tissue	MSP	Asian
Zhong, H.H., II	2017	15	15	75	11	116	Serum	MSP	Asian
Prasant, Y.	2017	28	0	32	60	120	Plasma	MSP	Asian
Sofia, S., I	2018	6	0	38	39	83	Plasma	q-MSP	Caucasian
Sofia, S., II	2018	108	1	29	26	164	Tissue	q-MSP	Caucasian

Tn = true negative; Tp = true positive; Fn = false negative; Fp = false positive; MSP = mehylation-specific PCR; q-MSP = quantitative mehylationspecific PCR;OS-MSP = the one-step methylation-specific polymerase chain reaction;MethyLight = high-throughput DNA methylation assay.

### Publication bias and quality assessment

No asymmetry was found in the Deek’s funnel plot and *P* > 0.05 indicated no publication bias (Supplementary Figure S1). The outcome of the QUADAS-2 (Supplementary Figure S2) showed high risk of bias on patient selection while low or unclear bias on index test, reference standard and flow and timing. So, the entire qualities of include studies were medium.

### TSA

The cumulative *Z*-curve (Supplementary Figure S3) crossed the monitoring boundaries and the number of samples (*n* = 3391) has exceed the required sample size (*n* = 411), confirming that our sample sizes were enough and the estimates are reliable.

### Diagnostic effect

**Figure 2:**
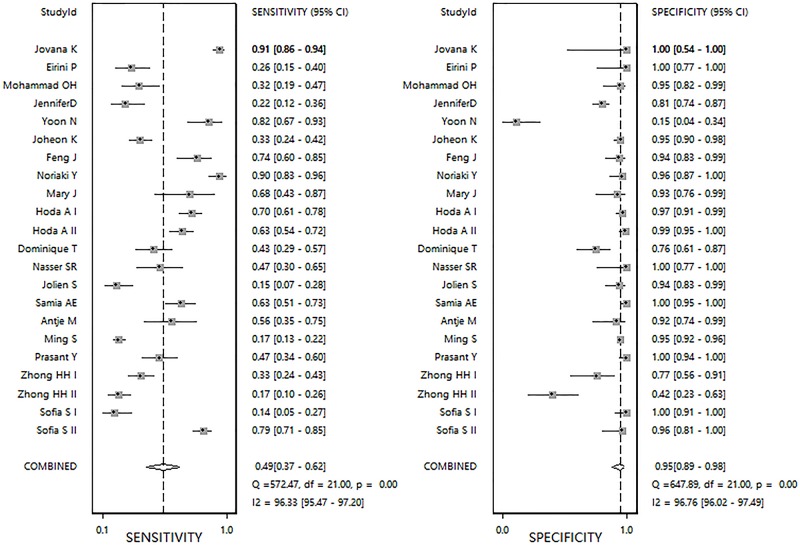
Forest plots of pooled sensitivity and specificity

**Figure 3:**
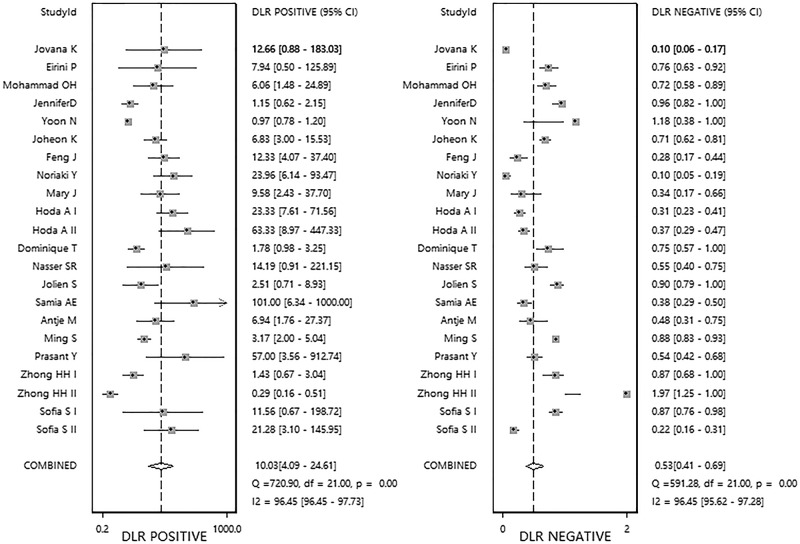
Forest plots of pooled positive likelihood ratio and negative likelihood ratio

**Figure 4:**
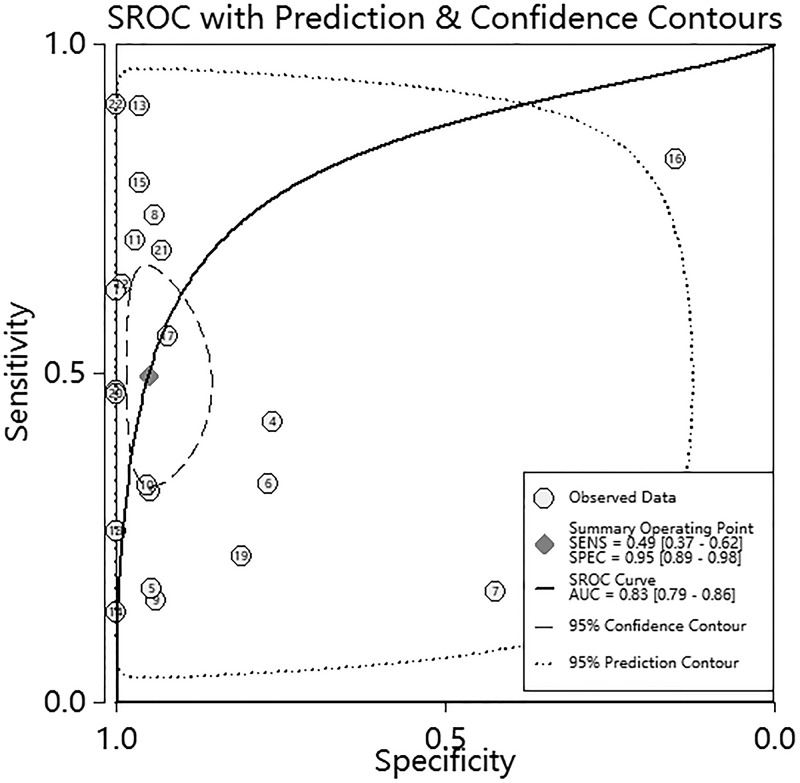
SROC curve for the diagnosis of RASSF1A methylation in breast cancer

The pooled sensitivity and specificity (Figure 2) of *RASSF1A* methylation were 0.49 (95%Cl 0.37–0.62) and 0.95 (95%Cl 0.89–0.98), respectively. The pooled PLR and NLR (Figure 3) were 10.03 (95%Cl 4.09–24.61) and 0.53 (95%Cl 0.41–0.69), respectively. The pooled DOR (Supplementary Figure S4) was 19.0 (95%Cl 7.00–54.00) and AUC (Figure 4) was 0.83 (95%Cl 0.79–0.86).

### Heterogeneity and subgroup analysis

In the threshold analysis, spearman correlation coefficient was –0.053 and *P*-value was 0.814, showing no threshold effect. Furthermore, a significant heterogeneity caused by non-threshold effect was seen. The result indicated that *I^2^* of sensitivity was 96.33%, *I^2^* of specificity was 96.76% and a random-effects model was utilized. To determine the sources of heterogeneity, we conducted subgroup analysis. For the ethnicity of BC patients, the pooled sensitivity, specificity, DOR and AUC of *RASSF1A* methylation to diagnosis Caucasian were 0.54 (0.38–0.69), 0.96 (0.85–0.99), 30.0 (7.0–124.0) and 0.84 (0.81–0.87), while the pooled sensitivity, specificity, DOR and AUC of *RASSF1A* methylation to detect other ethnic groups (Africa and Asian) were 0.43 (0.24–0.64), 0.94 (0.86–0.98), 10.0 (2.0–51.0) and 0.81 (0.77–0.84), respectively. For the sample size, the pooled sensitivity of *RASSF1A* methylation in articles with sample size <100 was 0.40 (0.24–0.60), specificity was 0.95 (0.77–0.99), DOR was 12.0 (3.0–44.0) and AUC was 0.72 (0.67–0.75), while the pooled sensitivity of *RASSF1A* methylation in articles with sample size ≥100 was 0.51 (0.35–0.67), specificity was 0.95 (0.89–0.98), DOR was 21.0 (5.0–80.0) and AUC was 0.87 (0.84–0.90), respectively. For the detection method, the pooled sensitivity of *RASSF1A* methylation detected by MSP was 0.64 (0.36–0.71), specificity was 0.95 (0.89–0.98), DOR was 21.0 (4.0–119.0) and AUC was 0.89 (0.82–0.94), while the pooled sensitivity of *RASSF1A* methylation detected by other methods (q-MSP, OS-MSP and MethyLight) was 0.57 (0.31–0.71), specificity was 0.93 (0.83–0.97), DOR was 14.0 (4.0–43.0) and AUC was 0.86 (0.82–0.88), respectively. For sample types, the pooled sensitivity of serum *RASSF1A* methylation was 0.55 (0.38–0.71) and specificity was 0.95 (0.85–0.98). DOR was 22.0 (7.0–74.0) and AUC was 0.86 (0.82–0.88), while the pooled sensitivity of *RASSF1A* methylation in other samples (tissue or plasma) was 0.40 (0.25–0.57), specificity was 0.95 (0.84–0.99), DOR was 14.0(2.0–88.0) and AUC was 0.74(0.70–0.78), respectively (Table 2).

**Table 2:** Subgroup analysis of diagnostic effect

Subgroup	No. of studies	Sample size	Sensitivity			Specificity			DOR (95%Cl)	AUC (95%Cl)
			Value (95%Cl)	*I^2^*	*P*	Value (95%Cl)	*I^2^*	*P*		
Overall	22	3391	0.49 (0.37–0.62)	96.33	0.00	0.95 (0.89–0.98)	96.76	0.00	19.0 (7.0–54.0)	0.83 (0.79–0.86)
Ethnicity										
Caucasian	14	1723	0.54 (0.38–0.69)	95.30	0.00	0.96 (0.85–0.99)	95.85	0.00	30.0 (7.0–124.0)	0.84 (0.81–0.87)
Others	8	1668	0.43 (0.24–0.64)	96.65	0.00	0.94 (0.86–0.98)	96.89	0.00	10.0 (2.0–51.0)	0.81 (0.77–0.84)
Sample size										
<100	7	448	0.40 (0.24–0.60)	90.18	0.00	0.95 (0.77–0.99)	95.19	0.00	12.0 (3.0–44.0)	0.72 (0.67–0.75)
≥100	15	2943	0.51 (0.35–0.67)	97.22	0.00	0.95 (0.89–0.98)	96.70	0.00	21.0 (5.0–80.0)	0.87 (0.84–0.90)
Detection method										
MSP	10	1276	0.64 (0.36–0.71)	95.86	0.00	0.96 (0.85–0.95)	92.49	0.00	21.0 (4.0–119.0)	0.89 (0.82–0.94)
Others	11	2063	0.57 (0.31–0.71)	97.61	0.00	0.93 (0.83–0.97)	98.08	0.00	14.0 (4.0–43.0)	0.86 (0.82–0.88)
Sample type										
Serum	8	1817	0.55 (0.38–0.71)	95.91	0.00	0.95 (0.85–0.98)	95.05	0.00	22.0 (7.0–74.0)	0.86 (0.82–0.88)
Others	14	1574	0.40 (0.25–0.57)	95.54	0.00	0.95 (0.84–0.99)	97.22	0.00	14.0 (2.0–88.0)	0.74 (0.70–0.78)

## Discussion

As an important tumor suppressor gene, *RASSF1A* has been reported as a new diagnostic biomarker for BC in numerous studies [8,9,12,13,32]. However, no comprehensive review had been performed to evaluate the diagnostic accuracy. This is the first diagnostic meta-analysis to assess the diagnostic value of *RASSF1A* methylation for BC.

The pooled sensitivity and specificity of *RASSF1A* methylation in our study was 0.49 and 0.95, respectively, which indicated that 49% of BC patients enjoyed high *RASSF1A* methylation levels, and 95% of non-breast-cancer patients had low *RASSF1A* methylation levels. We identified that *RASSF1A* methylation was a potential diagnostic biomarker for BC; but the sensitivity was low, thus it should be combined with other biomarkers to increase sensitivity. Additionally, the SROC curve and AUC can be made to assess diagnostic value, where AUC is closer to 1 signifies that the diagnostic method has better discrimination [33]. The AUC performed in the current meta-analysis was 0.83, showing that *RASSF1A* methylation is a helpful biomarker for BC diagnosis. The DOR was another indicator of diagnostic accuracy that comprehensively indicating sensitivity and specificity. The higher the value of DOR, the better the diagnosis effort was [34]. A DOR of 19.0 in our study displayed a favorable value of *RASSF1A* methylation. The PLR and NLR were other diagnostic indicators of clinical importance, PLR close to 10 and NLR close to 0 were thought more convincing to rule in or rule out disease, respectively [35]. In our research, the pooled PLR and NLR were 10.03 and 0.53 that have medium value of *RASSF1A* methylation.

Several DNA methylation genes were reported in BC; however, the most suitable diagnostic marker hasn’t been found yet [4,36]. The specificity of *RASSF1A* methylation was higher than others. But its sensitivity was lower. For example, Ming et al. showed that the specificity of *RASSF1A* methylation was 91.95%, while the specificity of *P16* methylation, *PCDHGB7* methylation, *SFN* methylation and *HMLH1* methylation were 84.32%, 54.66%, 38.98% and 77.97%, respectively [37]. The sensitivity of *RASSF1A* methylation was 17.16%, while the sensitivity of *P16* methylation, *PCDHGB7* methylation, *SFN* methylation and *HMLH1* methylation were 22.39%, 55.60%, 73.51% and 27.99%, respectively [9]. Given its low sensitivity, a combination with other gene methylation may upgrade the sensitivity and increase its utility in diagnostic test. Recently, Mohammad et al. showed that *RASSF1A* methylation combing *GSTP1* methylation and *RARβ2* methylation improved sensitivity for the diagnosis of BC (sensitivity: 62%, specificity: 87%). Kim et al. reported that the combination with Twist methylation, *RARβ* methylation and *RASSF1A* methylation could also improve sensitivity for the diagnosis of BC (sensitivity: 96%, specificity: 81%) [10,14–17,24].

The subgroup analysis was performed according to the ethnicity of BC patients, sample size, detection method and sample type. The pooled sensitivity, DOR and AUC of *RASSF1A* methylation diagnosing in Caucasian were 0.54, 30.0 and 0.84, while the pooled sensitivity, DOR and AUC of *RASSF1A* methylation in other ethnicities were 0.43, 10.0 and 0.81, respectively. The results indicated that Caucasian might be more susceptible to *RASSF1A* methylation. It suggested that genetic and environmental factors had impact on the diagnostic value of *RASSF1A* methylation [37]. When the sample size is expanded from <100 to ≥100, the pooled sensitivity (0.40 vs 0.51), DOR (12.0 vs 21.0) and AUC (0.72 vs 0.87) of *RASSF1A* methylation increased, suggesting that higher diagnostic value of *RASSF1A* methylation was generated after expanding the sample size. The pooled sensitivity, DOR and AUC of *RASSF1A* methylation detected by MSP were 0.64, 21.0 and 0.89, while the pooled sensitivity DOR and AUC of *RASSF1A* methylation detected by other methods were 0.57, 14.0 and 0.86, respectively. The results indicated that MSP was more effective in the detection of *RASSF1A* methylation. The reason of it might be that MSP and other methods required particular gene sequence information for the design of PCR primers. The different primers might have impacts on results [38]. The pooled sensitivity, DOR and AUC of serum *RASSF1A* methylation were 0.55, 22.0 and 0.86, while the pooled sensitivity, DOR and AUC of *RASSF1A* methylation in other sample types were 0.40, 14.0 and 0.74, respectively. The outcomes indicated that the detection of serum *RASSF1A* methylation was more suitable for diagnosing BC compared with tissue or plasma *RASSF1A* methylation.

Our meta-analysis has several disadvantages. First, all studies here were case–control studies, which have higher risks of bias. So more in-depth investigations with well-designed prospective cohort studies are needed. Second, some patients with multiple genes methylation included in the meta-analysis might have an impact on the generalizability of the findings. Third, we only reported one gene *RASSF1A* methylation in the present research and more candidate gene methylations should be excavated in the future. Fourth, the clinical heterogeneity and statistical heterogeneity might decrease the reliability of our outcomes. Fifth, we can’t provide our own experimental data for further verification. The following studies using both data from TCGA or laboratories are recommended to validate the result.

This is the first diagnostic meta-analysis investigating the accuracy of *RASSF1A* methylation for BC. Our evidence shows that *RASSF1A* methylation enjoys narrow applicability for diagnosing BC yet. To improve diagnostic accuracy of *RASSF1A* methylation, it should be combined with other biomarkers. The result may be influenced by ethnicity, detection method and sample type used. The detection of *RASSF1A* methylation in Caucasian is more susceptible, MSP was more effective in the detection of *RASSF1A* methylation and serum *RASSF1A* methylation was more suitable for diagnosing BC compared with tissue or plasma *RASSF1A* methylation. More well-designed prospective diagnostic studies are needed to confirm our results.

## Supporting information

**Supplementary Figure S1:** Deeks’ funnel plot for assessing publication bias.

**Supplementary Figure S2:** Quality assessments of included studies.

**Supplementary Figure S3:** Trial sequential analysis of the meta-analysis.

**Supplementary Figure S4:** Forest plots of pooled diagnostic odds ratio.

**Supplementary Table S1:** Search strategies of databases.

## Abbreviations

AUC: the area under the SROC curve
BC: breast cancer
DOR: diagnostic odds ratio
FN: false negative
FP: false positive
MSP: methylation-specific PCR
OS-MSP: the one-step methylation-specific polymerase chain reaction
q-MSP: quantitative methylation specific PCR
*RASSF1A*: RAS-association domain family 1 isoform A
TN: true negative
TP: true positive
