# Supplementary material for: Diagnostic value of RASSF1A methylation for breast cancer: a meta-analysis
Source: Biosci Rep. 2019 Jun 28;39(6):BSR20190923. doi: 10.1042/BSR20190923 (PMC6597854; doi:10.1042/BSR20190923)
Supplement: Supplementary file 1 [file bsr20190923_Supp1.pdf]

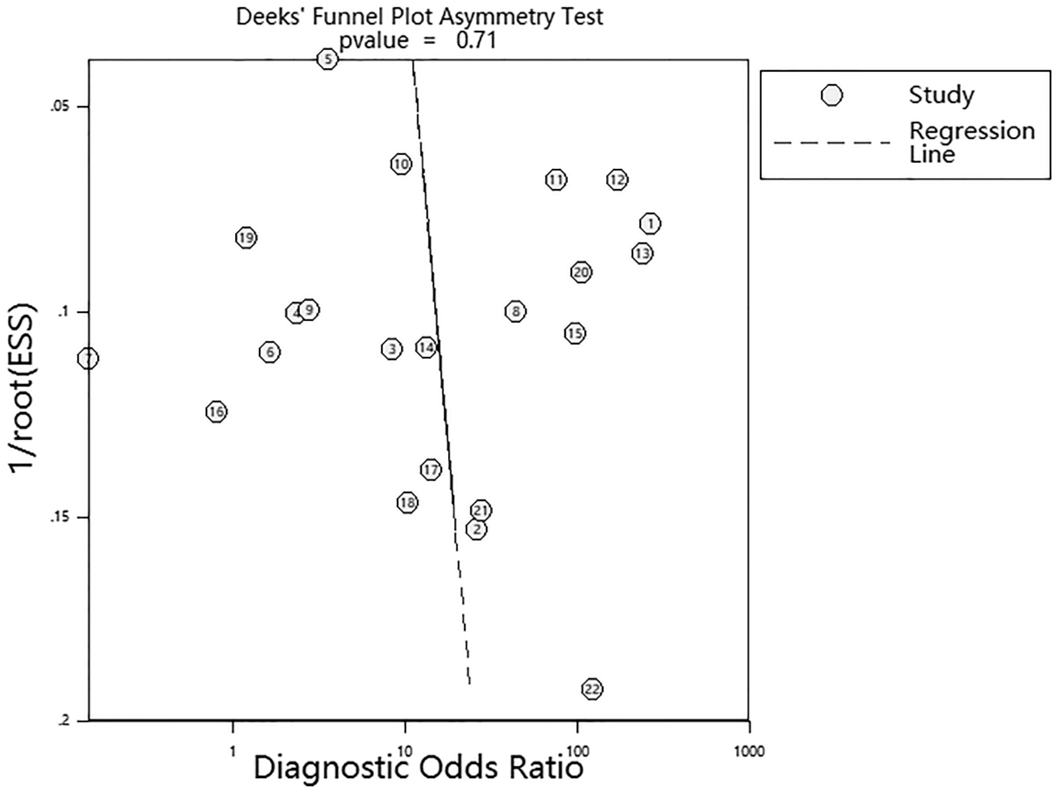

Supplementary Figure 1. Deeks’ funnel plot for assessing publication bias.

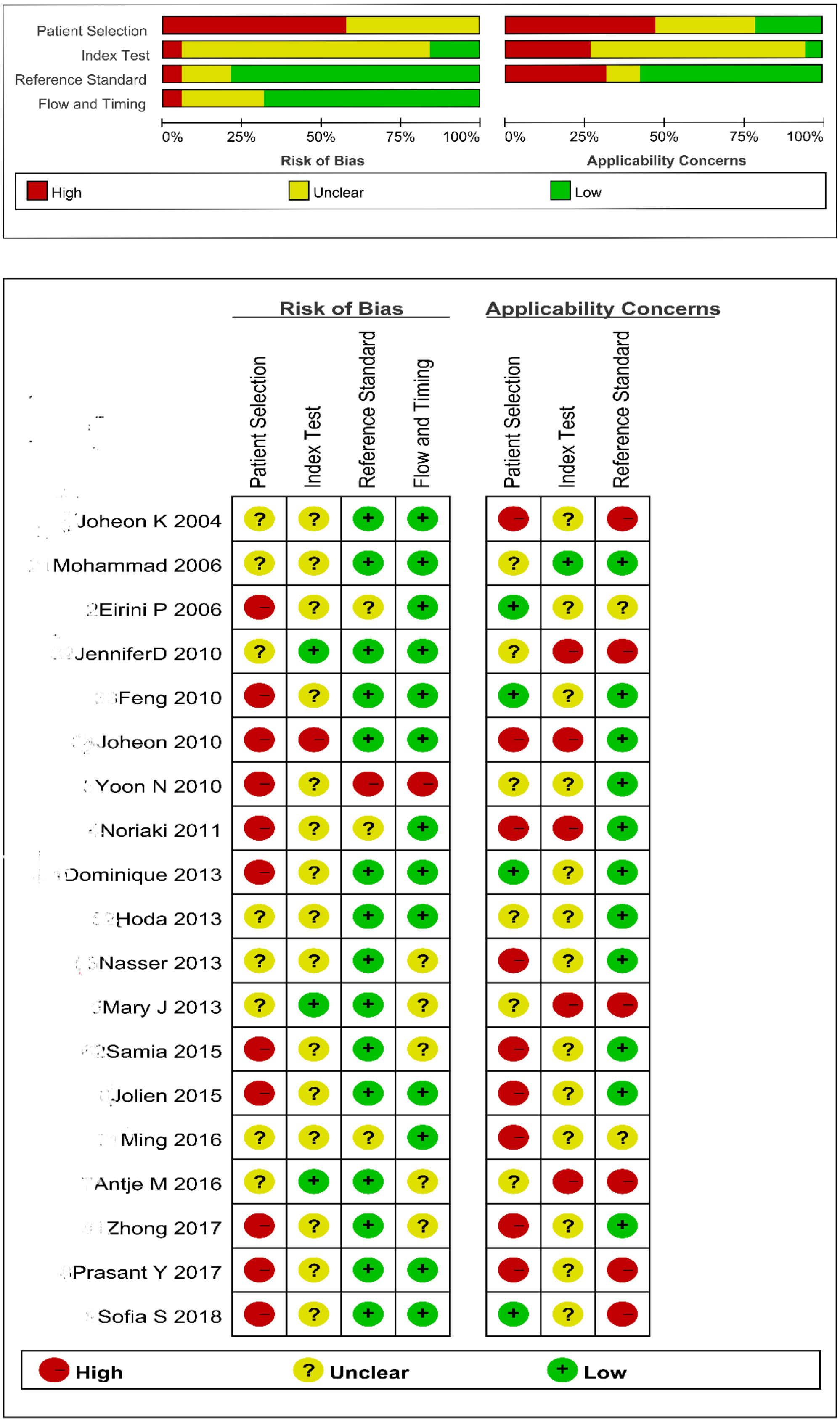

Supplementary Figure 2. Quality assessments of included studies.

Supplementary Figure 3 Trial sequential analysis of the meta-analysis.

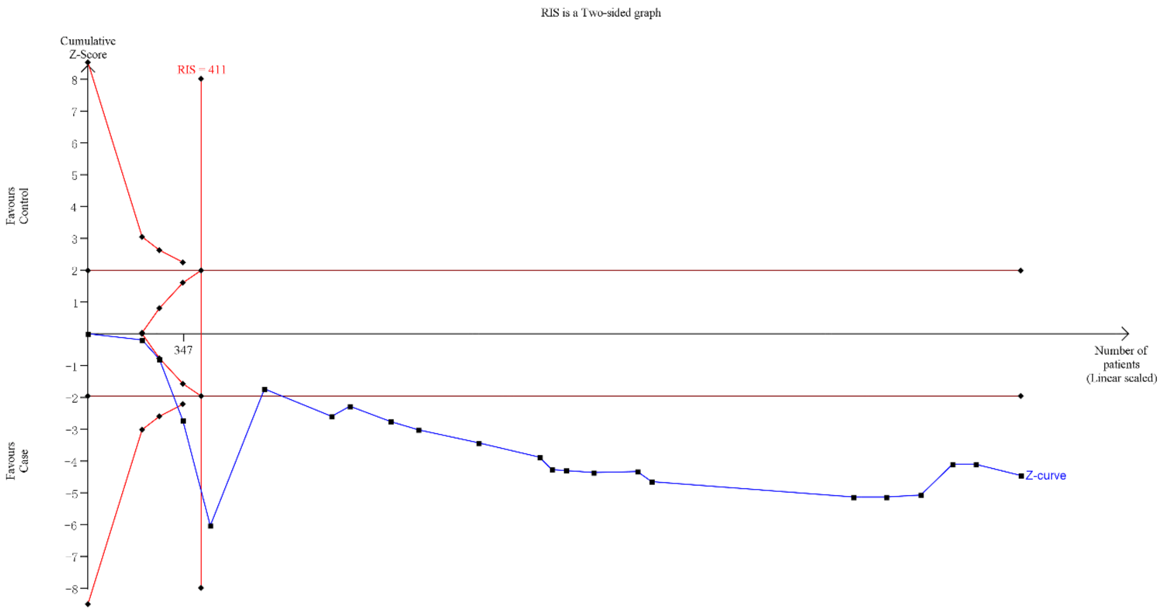

Supplementary Figure 3. Trial sequential analysis of the meta-analysis.

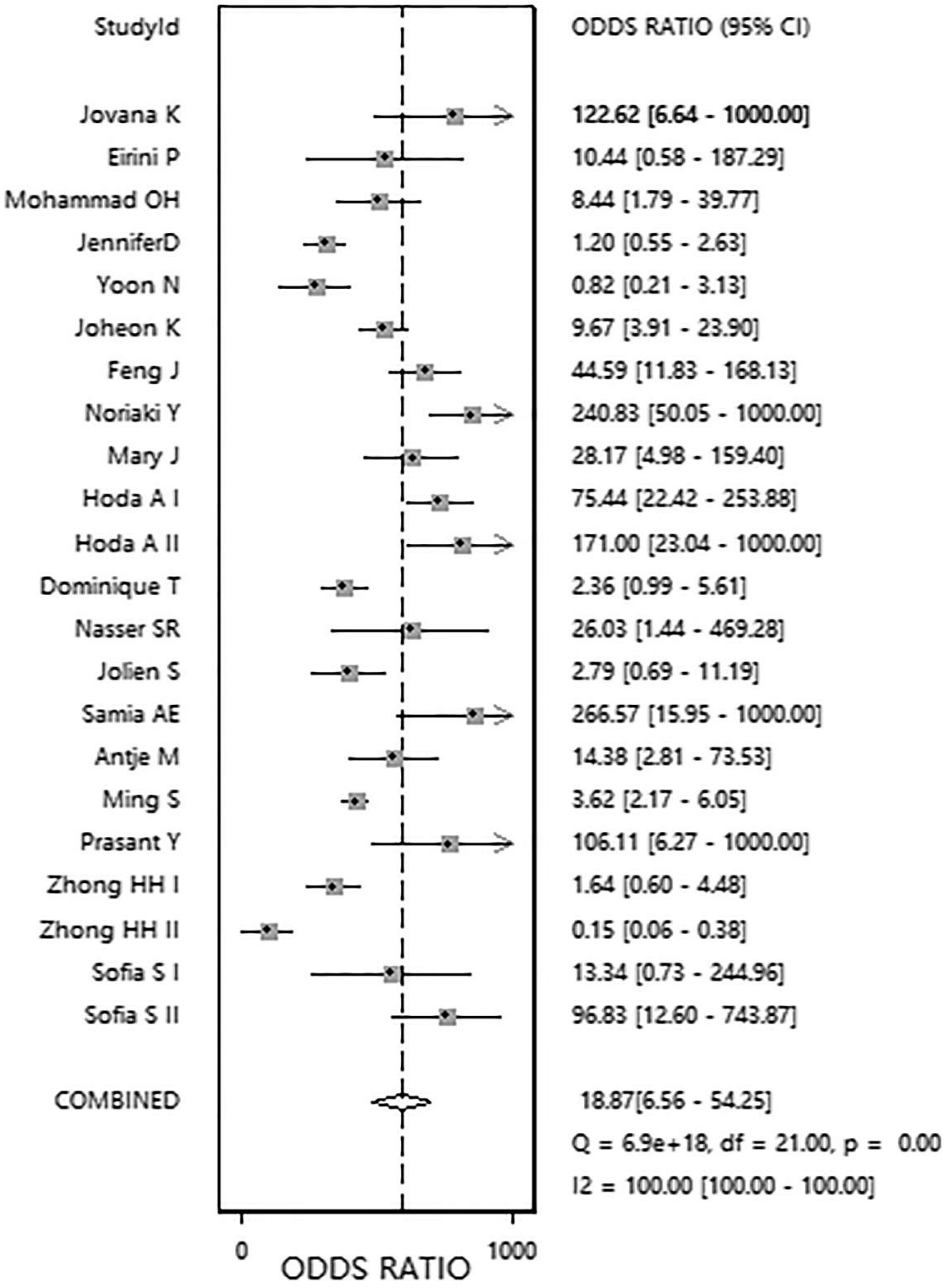

Supplementary Figure 4. Forest plots of pooled diagnostic odds ratio.

Supplementary Table 1. Search strategies of databases.

Databases	Search strategies	Search outcomes
Pubmed	#1 “breast neoplasms” [Mesh]<br>#2 Search (((((((((((((((((((((((((((((((((((((Breast Neoplasm) OR Neoplasm, Breast) OR Breast Tumors) OR Breast Tumor) OR Tumor, Breast) OR Tumors, Breast) OR Neoplasms, Breast) OR Breast Cancer) OR Cancer, Breast) OR Mammary Cancer) OR Cancer, Mammary) OR Cancers, Mammary) OR Mammary Cancers) OR Malignant Neoplasm of Breast) OR Breast Malignant Neoplasm) OR Breast Malignant Neoplasms) OR Malignant Tumor of Breast) OR Breast Malignant Tumor) OR Breast Malignant Tumors) OR Cancer of Breast) OR Cancer of the Breast) OR Mammary Carcinoma, Human) OR Carcinoma, Human Mammary) OR Carcinomas, Human Mammary) OR Human Mammary Carcinomas) OR Mammary Carcinomas, Human) OR Human Mammary Carcinoma) OR Mammary Neoplasms, Human) OR Human Mammary Neoplasm) OR Human Mammary Neoplasms) OR Neoplasm, Human Mammary) OR Neoplasms, Human Mammary) OR Mammary Neoplasm, Human) OR Breast Carcinoma) OR Breast Carcinomas) OR Carcinoma, Breast) OR Carcinomas, Breast) Sort by: [pubsolr12]<br>#3 (#1 OR #2)<br>#4 Search (((RASSF1A methylation) OR RASSF1A hypermethylation) OR RASSF1A DNA methylation) OR RASSF1 protein, human Sort by: [pubsolr12]<br>#5 (#3 AND #4)<br>Filters: Publication date to 2019/5/28 AND Language: English	244
Web of science	Topic=(RASSF1A methylation OR RASSF1A DNA methylation OR RASSF1A protein, human OR RASSF1A hypermethylation OR RASSF1A DNA hypermethylation) AND (Breast Neoplasm OR Neoplasm, Breast OR Breast Tumors OR Breast Tumor OR Tumor, Breast OR Tumors, Breast OR Neoplasm*, Breast OR Breast Cancer OR Cancer, Breast OR Mammary Cancer OR Cancer, Mammary OR Cancers, Mammary OR Mammary Cancer* OR Malignant Neoplasm of Breast OR Breast Malignant Neoplasm OR Breast Malignant Neoplasms OR Malignant Tumor of Breast OR Breast Malignant Tumor OR Breast Malignant Tumor* OR Cancer of Breast OR Cancer of the Breast OR Mammary Carcinoma, Human OR Carcinoma, Human Mammary OR Carcinoma*, Human Mammary OR Human Mammary Carcinoma* OR Mammary Carcinoma*, Human OR Human Mammary Carcinoma OR Mammary Neoplasms, Human OR Human Mammary Neoplasm OR Human Mammary Neoplasms OR Neoplasm, Human Mammary OR Neoplasm*, Human Mammary OR Mammary Neoplasm, Human OR Breast Carcinoma OR Breast Carcinoma* OR Carcinoma, Breast OR Carcinoma*, Breast）	647

	Filters: Timespan up to 2019 AND Search language = English	
The Cochrane Library	#1 MeSH descriptor: [Breast Neoplasms] explode all trees<br>#2 breast cancer OR breast neoplasm OR breast carcinoma OR breast malignant tumor OR mammary cancer OR mammary neoplasm OR mammary carcinoma OR mammary malignant tumor<br>#3 (#1 OR #2)<br>#4 RASSF1A methylation OR RASSF1A DNA methylation OR RASSF1A protein, human OR RASSF1A hypermethylation OR RASSF1A DNA hypermethylation<br>#5 (#3 AND #4)	2
